# Clinical report of metacarpal melorheostosis: a rare disease with “the dripping candle wax” appearance on different imaging modalities

**DOI:** 10.1093/omcr/omae115

**Published:** 2024-10-10

**Authors:** Oumaima Mesbah, Manal Jidal, Rachida Saouab, Jamal El Fenni

**Affiliations:** Department of Radiology, Mohammed V Military Instruction Hospital, Faculty of Medicine and Pharmacy of Rabat, Mohamed V University, Rabat 91000, Morocco; Department of Radiology, Mohammed V Military Instruction Hospital, Faculty of Medicine and Pharmacy of Rabat, Mohamed V University, Rabat 91000, Morocco; Department of Radiology, Mohammed V Military Instruction Hospital, Faculty of Medicine and Pharmacy of Rabat, Mohamed V University, Rabat 91000, Morocco; Department of Radiology, Mohammed V Military Instruction Hospital, Faculty of Medicine and Pharmacy of Rabat, Mohamed V University, Rabat 91000, Morocco

**Keywords:** melorheostosis, bone, standard X-ray, CT, MRI

## Abstract

Melorheostosis is a rare benign bone dysplasia characterized by dysostosis and sclerosis. The classic “dripping candle wax” appearance on imaging is a typical finding for the diagnosis. The authors report the case of a patient presenting with a hard and painful mass on the dorsal side of the hand.

## Introduction

Melorheostosis or Leri disease is a rare benign sclerosing bone dysplasia, which typically affects both cortical bone and the surrounding soft tissue structures in a sclerotomy distribution. The origin of its name comes from Greek (“melos” = “member”, “rhein” = “flow”, “ostos” = “bone”) [1]. In the majority of cases, bone abnormalities follow a monomelic and linear metameric distribution; with preferential involvement of the lower limb. The characteristic feature of this disorder is the irregular and uneven distribution of cortical bone along the affected bones, resembling candle wax dripping. This distinctive radiographic appearance is key diagnostic criteria [2].

## Case report

**Figure 1:**
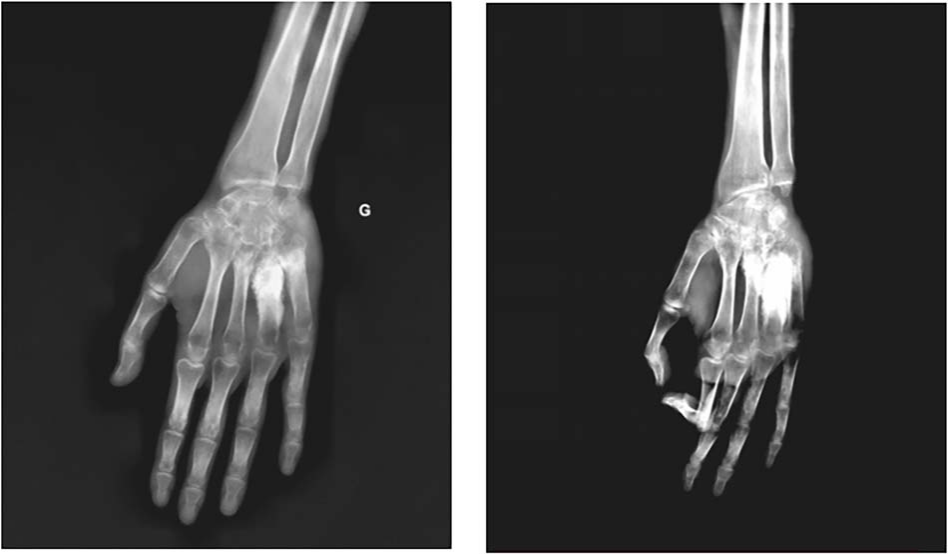
Radiographs of the left hand front (**a**) and oblique (**b**), showing cortical hyperdense lesion of the 3rd, 4th and 5th metacarpal bone, accentuated at the level of the 4th metacarpal with obliteration of the medulla, with a “dripping candle wax” appearance.

**Figure 2:**
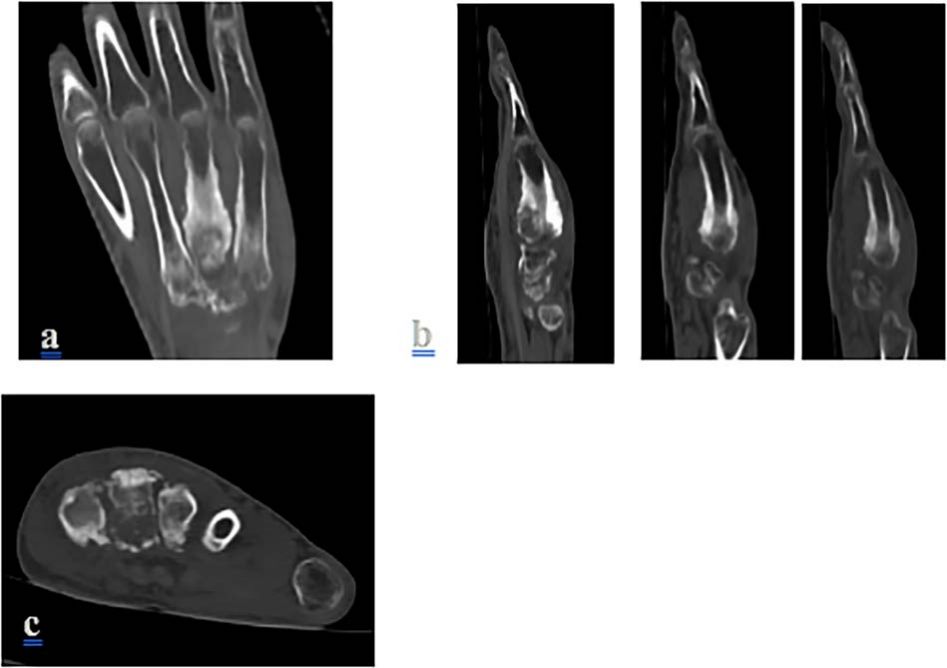
CT reconstruction of the coronal (**a**), sagittal (**b**) and axial image (**c**) showing cortical hyperostosis and sclerosis of the 3rd, 2nd and 5th metacarpal bones.

**Figure 3:**
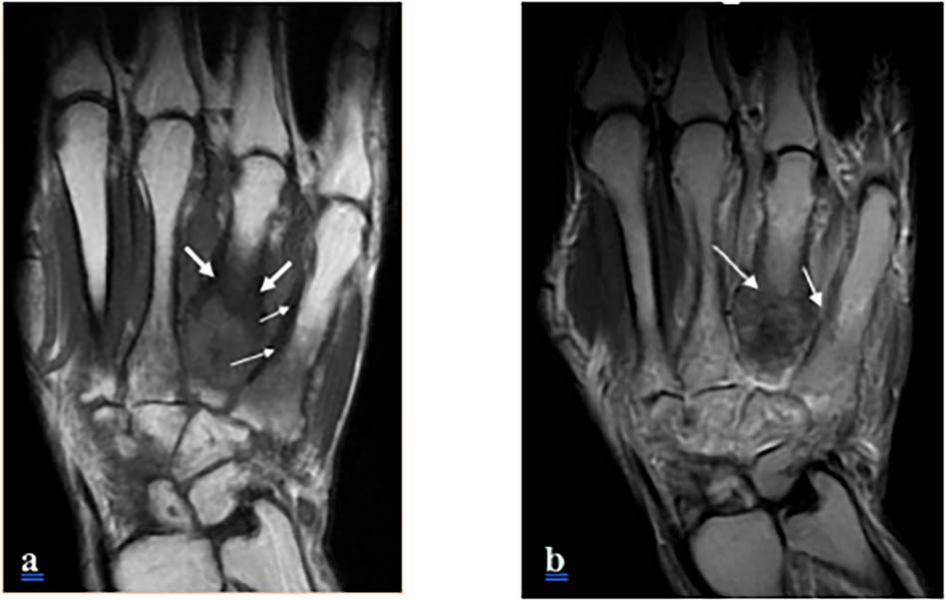
MRI of the left hand, a coronal section, on T1 (**a**) and T2 (**b**) weighted sequences, showing cortical thickening of the 4th and 5th metatarsal, as a hyposignal and isosignal on all sequences.

**Figure 4:**
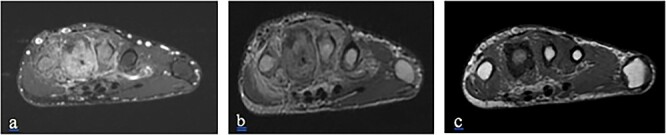
MRI of the left hand, an axial section, on T1weighted sequence (**c**) and T1with fat saturation (**b**) sequences, showing cortical thickening of the 3rd, 4th and 5th metatarsal, as a hypointense signal, enhanced after injection (**a**) with infiltration of the surrounding soft tissue.

A 23-year-old young man, with no family or personal past history, presented to the orthopedic clinic for painful swelling of the dorsal side of his left hand, which had been developing for three years. Standard radiography was offered and revealed periosteal cortical thickening to the 3rd, 4th and 5th metacarpus, obliterating the medullary canal. at the 4th metacarpal, extending along the lateral and medial sides of the bone, creating a “candle wax dripping” appearance (Fig. 1) On computed tomography (CT), we note the continuous aspect of the hyperostosis at the level of the base and the body of the 4th metacarpal bone, as well as at the base of the 3rd and 5th metacarpal (Fig. 2) On magnetic resonance imaging (MRI), the lesions appear hypointense with some spots in asignal on all sequences as well as Centro medullary contrast enhancement along the cortico-periosteal hyposignal (T1, T2 and in proton density with fat saturation). [DP FAT SAT]) with inflammatory reaction of adjacent muscular structures and soft tissues. (Figs 3 and 4).

## Discussion

Melorheostosis is a developmental anomaly that belongs to the groups of sclerosing bone dysplasias. Leri and Joanny described this disease for the first time since 1922 as hyperostosis in a “dripping candle wax” sign [1]. All ages can be affected (popular age range: 2 to 64 years) without sex predominance [3]. It manifestations are often discreet until adolescence.

Melorheostosis most often affects the long bones of the limbs and the auxiliary skeleton. It is sometimes found in the bones of the hands and feet, but rarely in the axial skeleton [4].

The origin of melorheostosis has not yet been clarified and many theories have been proposed. Murray and McCredie [5] hypothesize that one of the causes is a segmental sensory lesion of neural crest Fig. 3 occurring during embryogenesis. It was proven by Hellemans et al. [5] that melorheostosis is the result of a loss-of-function mutation of LEMD3 on chromosome 12q. Other study groups have demonstrated several genes codes for a nuclear protein that normally inhibits both transforming growth factor and bone morphogenic protein leading to melorheostosis which opens the prospect of treatment by gene therapy in the future [5].

Bone dystrophy can be asymptomatic, discovered incidentally or present as deformities, pain, stiffness or limitation of joint mobility. When the surrounding soft tissues are also involved, a pattern of cutaneous scleroderma involving edema, hypertrichosis, subcutaneous fibrosis, fibroids, or fibrolipomas may occur.

The diagnosis is often easily suggested by the typical appearance on imaging. On standard radiography, melorheostosis appears as linear hyperostosis, characteristic of “candle wax dripping”. The distrubition is monomelic, single or multi-focal, with a massive increase in the compact bone tissue of the cortices, causing a narrowing of the medulla. (Fig. 1). CT and MRI are generally not necessary for diagnosis except in complex cases. CT shows sclerotic bone lesions distorting the cortical outline of the bone and allows for a more precise loco regional analysis (Fig. 2). MRI makes it possible to evaluate bone deformation with a more precise analysis of the extension to the soft tissues, search for associated vascular or nervous anomalies and eliminates possible differential diagnoses. The lesions typically appear in hyposignal and do not usually change after injection, however an increase in signal after injection, a characteristic found in our observation, (Figs 3 and 4) suggest the immature character of the lesion with the presence of vascular-fibrous reactions around the sites of bone proliferation [6].

Bone scintigraphy is an imaging investigation with an important role in highlighting bone metabolic activity; it has been used in the process of diagnosing patients with suspected melorheostosis [7] The scintigraphic characteristic of melorheostosis is moderate uptake in the late phases [2].

Image findings can have a range of different manifestations and should be distinguished from other lesions like osteosarcoma, osteoid osteoma, parosteal osteosarcoma, osteochondroma, osteopetrosis, osteomyelitis, osteopoikilosis, osteopathy, fibrous dysplasia and myositis ossificans [8].

A variety of surgical and conservative treatments have been used for melorheostosis in an attempt to relieve pain and improve quality of life. Conservative treatments include bisphosphonates and nonsteroidal anti-inflammatory drugs [9]. Other conservative treatments include physical therapy, brace, serial casting, sympathectomies, and nerve block [10]. In many cases, melorheostosis requires surgical treatment such as soft tissue procedures including tendon lengthening, capsulotomy, fasciotomy, excision of fibrous and osseous tissue [11].

## Conclusion

Melerheoestosis is a rare benign bone disease with a classic characteristic “dripping candle wax” radiological appearance allowing the diagnosis to be made. The findings in our case were characteristic of the disease on different imaging modalities. Our patient refused conservative surgery and was given nonsteroidal anti-inflammatory drugs. The lesion stayed stable in further follow ups.

Given the limited number of cases worldwide and thus, the lack of clinical guidelines regarding specialized treatment, melorheostosis remains an incompletely understood disease.

In the case of asymptomatic patients, conservative treatment seems to be a good option with optimal results. Patients with severe symptoms and significantly limited joint mobility should benefit from surgery for both curative and quality-of-life purposes.

## References

[1] Leri A, Joanny J. Une affection non decrite des os: hyperostose « en coulee » sur toute la longueur d'un membre ou « melorheostose ». Bull Mem Soc Med Hosp Paris 1922;46:1141–5.

[2] Motimaya AM, Meyers SP. Melorheostosis involving the cervical and upper thoracic spine: radiographic, CT, and MR imaging findings. AJNR Am J Neuroradiol 2006;27:1198–200. 16775263 PMC8133940

[3] Kotwal A, Clarke B. Melorheostosis: a rare sclerosing bone dysplasia. Curr Osteoporos Rep 2017;15:335–42. 28676968 10.1007/s11914-017-0375-y

[4] Fick C, Fratzl-Zelman N, Roschger P. et al. Melorheostosis: a series of clinical, pathological and radiological cases. Am J Surg Pathol 2019;43:1554–9. 31386640 10.1097/PAS.0000000000001310 PMC7832124

[5] Hoang VT, Van HAT, Chnsmphou V. et al. Trinh: the dripping candle wax sign of melorheostosis. SAGE Open Med Case Rep 2020;8:2050313X20940564. 10.1177/2050313X20940564. PMC7446549 32922791

[6] El Mhabrech H, Gamaoun W, Arifa N. et al. La mélorhéostose: à propos d'un cas radio clinique. J Radiol 2007;88:397–400. 17457273 10.1016/s0221-0363(07)89838-6

[7] Izadyar S, Gholamrezanezhad A. Bone scintigraphy elucidates different metabolic stages of melorheostosis. Pan Afr Med J 2012;11:21. 22514755 PMC3325059

[8] Wordsworth P, Chan M. Melorheostosis and osteopoikilosis: a review of clinical features and pathogenesis. Calcif Tissue Int 2019;104:530–43. 10.1007/s00223-019-00543-y. 30989250

[9] Jain V, Arya R, Bharadwaj M. et al. Melorheostosis: Clinicopathological features, diagnosis, and management. Orthopedics 2009;32:512–20. 19634844 10.3928/01477447-20090527-20

[10] Zhang C, Dai W, Yang Y. et al. Melorheostosis: Clinicopathological features, diagnosis, and management. Intraitable Rare Dis Res 2013;2:51–4. 10.5582/irdr.2013.v2.2.51. PMC4204579 25343102

[11] Ethunandan M, Khosla N, Tilley E. et al. Melorheostosis involving the craniofacial skeleton. J Craniofac Surg 2004;15:1062–5. 15547407 10.1097/00001665-200411000-00038

